# Exploring functional metabolites and proteomics biomarkers in late-preterm and natural-born pigs

**DOI:** 10.3389/fvets.2024.1340849

**Published:** 2024-04-24

**Authors:** Jie Chong, Juan Wang, Hang Zhong, Jinwei Zhang, Yuchun Ding, Liangpeng Ge, Jideng Ma, Jing Sun

**Affiliations:** ^1^Chongqing Academy of Animal Sciences, Chongqing, China; ^2^Farm Animal Genetic Resources Exploration and Innovation Key Laboratory of Sichuan Province, Sichuan Agricultural University, Chengdu, China; ^3^National Center of Technology Innovation for Pigs, Chongqing, China

**Keywords:** delivery model, cesarean sections, natural delivery, newborn pigs, SCFAs, organic acids, fatty acid metabolism pathway

## Abstract

**Introduction:**

Pigs are often used to study the intestinal development of newborns, particularly as preterm pig models that mimic the intestinal growth of human preterm infants. Neonatology’s study of delivery mode’s impact on neonatal development is crucial.

**Methods:**

We established 14 newborn pigs delivered via cesarean sections (C-section, at 113 days of gestational age, CS group) and 8 naturally born pigs were used as controls (at 114 days of gestational age, NF group). The impact of two alternative delivery procedures (C-section and natural birth) on the levels of short-chain fatty acids (SCFAs) and organic acids in the hepatic and intestines of newborn pigs were compared using metabolomics. The underlying molecular pathways are examined at the “protein-metabolite” level by integrating proteomic data.

**Results:**

The findings demonstrated that the mode of delivery changed the metabolism of SCFAs in newborn pigs, perhaps by affecting the physiology levels of cyclic intermediates such as lactate and malate in the pyruvate metabolic pathway. Additionally, by participating in the fatty acid metabolism pathway, two distinct proteins (FASN and HSD17B4) may impact the physiological concentration of these tiny metabolites.

**Discussion:**

In conclusion, this study provided reliable animal model data for understanding the physiological SCFA metabolic information and its affecting mechanism of large-gestational age preterm infants.

## 1 Introduction

Annually, 15 million preterm infants are delivered before 37 weeks of gestation, with an average mortality rate of 10 percent (1). Preterm newborns are more vulnerable to illness and maybe even death due to physiological reasons such as their fragile bodies and immature organs (2, 3). Studies have found that preterm infants have a higher mortality rate, poor postnatal growth, sepsis, chronic lung disease, necrotizing enterocolitis (NEC), retinopathy of prematurity, and poor abnormal neurodevelopmental outcomes (4). Preterm newborns are primarily classified as very preterm, early preterm, moderate preterm, late preterm, and early term based on gestational age (5, 6). About 80% of preterm births occur late in pregnancy, and these infants are more likely to die or have chronic illnesses including metabolic or respiratory disorders (7–9). Compared to full-term infants, late preterm and early-term infants have greater rates of short- and long-term morbidity and mortality (10). The more concerning fact is that the increasing incidence of cesarean sections (C-section) (19.1% worldwide and 25% in Europe) complicates matters further for preterm infants’ survival and normal development (11, 12). Research has shown that different birth modes affect the early colonization of intestinal microorganisms. C-sections result in a decrease in the richness and diversity of intestinal microbiota, including a decrease in *Bacteroides* species and a decrease in the carry quantity and density of *Streptococcus pneumoniae* (13–15). Transferring the vaginal microbiota to newborns may aid in the recovery of metabolic disorders brought on by C-section (16). In addition, the birth mode also affected the anti-pneumococcal antibody response, and natural delivery was positively associated with certain beneficial bacteria (17). Overall, the birth mode plays a crucial role in maintaining the health of newborn infants.

As biomarkers and mediators of diseases, metabolites play a crucial role in host health, which has attracted wide attention (18, 19). Metabolites, which can stimulate gastrointestinal motility and alter intestinal transport time, impact intestinal homeostasis and energy balance (20, 21). Endogenous metabolites, microbial metabolites, and non-endogenous metabolites derived from external metabolites are the three categories of metabolites found in living organisms (22). SCFAs are a group of saturated fatty acids that possess a chain length ranging from one to six carbon atoms. These compounds are primarily generated in the colon through the fermentation of dietary fiber. Depending on the amount of fiber in the diet, the estimated daily production rate of SCFAs in the gut is between 500 and 600 mmol (23). Butyrate (4 carbons), propionate (3 carbons), and acetate (2 carbons) are the most prevalent SCFAs produced (24). SCFAs play a vital role in immune regulation and serve as an energy source for the host, providing energy to the colon epithelium (25, 26). They have various biological functions, including regulating the activity of mucosal immune cells, protecting the intestinal epithelium, lowering colon pH, preventing the growth of harmful bacteria, and regulating the metabolism of energy, inflammation, and tumor growth and development (27). Following absorption, SCFAs contribute to the host’s energy metabolism through the mitochondria’s citric acid cycle (CAC) (28). The CAC, which is the central hub of cellular metabolism and where nutrients are oxidized to produce energy and biosynthetic intermediates, involves several key intermediate metabolites, including organic acids like pyruvate, fumarate, succinate, malate, oxaloacetate, α-AKG, citrate, lactate, oxalic acid, etc. (29). Large molecules like nucleotides, lipids, and proteins are biosynthesized with the help of these intermediary metabolites.

Animal testing is an essential scientific procedure that helps get innovative treatments from the lab to the patient (30). It advances our knowledge of the disease process, offers insightful information on the efficacy and safety of potential treatments, and is mandated by regulatory bodies to guarantee the safety of human subjects (31). Even if there are initiatives to use fewer animals in research, animal testing is still a necessary tool until more effective alternatives are created (32). In particular, preterm pig models have shown to be very useful for studying the onset, progression, and therapy of illnesses as well as the physiological development of preterm infants (33). Late preterm infants are the highest percentage of preterm infants and deserve special attention. However, the current studies mainly focused on very preterm infants or early preterm infants, and the research on animal models of late preterm infants is limited (34). Therefore, the goal of this study was to create a group of late preterm newborn pigs (at 113 gestational age) by C-section (6), with full-term newborn pigs (at 114 gestational age) born naturally as the control. To examine the impact of birth mode on the metabolic profiles of SCFAs and organic acids in the intestine and liver of newborn pigs, and analyze the possible pathways. The expected results of this study not only have positive implications for sow delivery management but may also help provide reliable animal data results for strategies to reduce preterm and early-term infants.

## 2 Materials and methods

### 2.1 Ethics approval

The institutional ethics committee of the Chongqing Academy of Animal Sciences (Chongqing, China) reviewed the relevant ethical issues and approved this study (permit number xky-20150113). All of the experiments were conducted at the Experimental Swine Engineering Center of the Chongqing Academy of Animal Sciences (CMA No. 162221340234; Chongqing, China).

### 2.2 Animal and experimental design

According to the local standard “Technical Specification for Germ-free (GF) Pig Production” (DB50/T 1043-2020) of Chongqing Municipality, pregnant sows used in this study were prepared for experimental usage. The three parturient sows that bred with the same boar were selected from the closed herd of self-breeding. They were not immunized, and five vertical transmission viruses—African swine fever virus, classical swine fever virus, porcine reproductive and respiratory syndrome virus, porcine pseudorabies virus, and porcine parvovirus—were all excluded. The pregnant sows were kept on a typical diet until they gave birth. The pregnant sow underwent C-section operations one day before the expected delivery (at 113 gestational age), and 14 newborn pigs were acquired as the experimental group (CS group). Eight newborn pigs delivered naturally (at 114 gestational age) served as the control group (NF group). Newborn pigs were placed under a thermal lamp until anesthetized and sampled.

### 2.3 Sample collection

Piglets were given respiratory anesthesia with isoflurane. Their anterior venous blood samples were sampled while they were under anesthesia, and the abdominal cavity was subsequently opened to get blood samples from the portal vein. These blood samples are kept and used for routine blood parameters and targeted metabolomics assay. The pigs were put to death by bloodletting while under anesthesia, and tissue samples of the liver and intestinal segments (duodenum, jejunum, ileum, cecum, and colon) were immediately taken from the animals, frozen in liquid nitrogen, and stored at −80°C for real-time quantitative PCR and proteome analysis. For the study of metabolites such as SCFAs and organic acids, the intestinal contents (not less than 500 mg/intestinal segment) taken from the intestinal lumen of the jejunum, ileum, cecum, colon, and rectum (or fresh feces) were collected on ice and then immediately frozen at −80°C.

### 2.4 Blood parameters measurement

A total of 23 routine blood indexes were tested in this study, including white blood cell count (WBC, 10^9^/L), neutrophil count (NEU, 10^9^/L), neutrophil ratio (NEU%), lymphocyte count (LYM, 10^9^/L), lymphocyte ratio (LYM%), and monocyte count (MON, 10^9^/L), monocyte ratio (MON%), eosinophil count (EOS, 10^9^/L), eosinophil ratio (EOS%), basophil count (BAS, 10^9^/L), basophil ratio (BAS%), red blood cell count (RBC, 10^12^/L), hemoglobin concentration (HGB, g/L), hematocrit (HCT, %), mean corpuscular volume (MCV, fL), mean corpuscular hemoglobin content (MCH, pg), mean corpuscular hemoglobin concentration (MCHC, g/L), red blood cell distribution width variation width (RDW-C, %), red blood cell distribution width standard deviation (RDW-S, fL), platelet count (PLT, 10^9^/L), mean platelet volume (MPV, fL), platelet distribution width (PDW, fL), and platelet crit (PCT, %). A total of 19 blood biochemical indicators were tested in this study, and they are alanine aminotransferase (ALT, U/L), aspartate aminotransferase (AST, U/L), glutamyl aminotransferase (GGT, U/L), alkaline phosphatase (ALP, U/L), aspartate aminotransferase/alanine (AST/ALT), total protein (TP, g/L), albumin (ALB, g/L), globulin (GLO, g/L), globulin (A/G), cholinesterase (CHE, U/L), blood urea nitrogen (BUN, mmol/L), creatinine (CREA, μmol/L), uric acid (UA, mmol/L), total cholesterol (CHOL, mmol/L), triglyceride (TG, mmol/L), glucose (GLU, mmol/L), potassium (K, mmol/L), sodium (NA, mmol/L), and chloride (CL, mmol/L). The above blood physiological and biochemical indexes were tested by Yongrong Hospital, Rongchang District, Chongqing.

## 3 Targeted metabolomics assays

### 3.1 Sample preparation

Samples were thawed at room temperature and mixed for 10 s. 50 μL was taken, 150 μL of methanol was added and vortexed for 5 min. After centrifugation at 13,200 r/min, 50 μL of the supernatant was removed, 50 μL of the internal standard was added, 100 μL of the derivative reagent was added, incubated for 30 min at room temperature, 50 μL of the protective agent was added, and 750 μL of water was added and vortexed for 10 s. The samples were centrifuged at 13,200 r/min for 10 min and the supernatant was removed for detection. The standards were subjected to the same derivations. Pre-treatment of tissues and feces: samples of a certain mass were weighed and homogenized, then 500 μL of water and 500 μL of methanol were added and extracted by vortex for 30 min. 50 μL of the supernatant was centrifuged at 13,200 r/min, 50 μL of the internal standard was added, 100 μL of the derivative reagent was added, incubated for 30 min at room temperature, 50 μL of the protective agent was added, and 750 μL of water was added, vortexed for 10 s. The samples were centrifuged at 13,200 r/min for 10 min and the supernatant was removed for detection. The standards were subjected to the same derivations. Next, the supernatant was collected to conduct subsequent analyses.

### 3.2 LC–MS/MS analysis

The LC–MS/MS analysis was conducted using the UPLC I-CLASS system (Waters Corporation, Milford, MA, United States) in combination with the Xevo TQ-S Microsystem (Waters Corporation, Milford, MA, United States). Metabolites were separated by chromatography using the BEH C8 column (100 × 2.1 mm, 1.7 μm, Waters Corporation, Milford, MA, United States). Water containing 0.1% (v/v) formic acid and carbinol were used as mobile phases A and B, respectively. Chromatographic elution settings included 0 min, 10% B; 2 min, 20% B; 9 min, 55% B; 10 min, 100% B; 11 min, 100% B; 11.1 min, 10% B; 13 min, 10%B. Next, the flow rate, column temperature, as well as injection volume, were predetermined at 20 L/Hr, 45°C, and 4 μL, respectively.

### 3.3 Metabolomics data analysis

Targeted metabolic profiling of liver, colon contents, feces, and blood samples by liquid chromatography–tandem mass spectrometry (LC–MS/MS) was performed to analyze metabolic profiles. LC–MS/MS was used to quantify 17 metabolites, including 9 organic acids and 8 SCFAs. The SCFAs measured included acetate, propionate, isobutyrate, butyrate, valerate, isovalerate, caproate, and heptanoic acid, and the organic acids measured included pyruvate, fumarate, succinate, malate, oxaloacetate, α-AKG, citrate, and lactate and oxalic acid. OPLS-DA was conducted on the data obtained from the metabolite profiling to discriminate between groups. With the OPLS-DA model, the Variable Importance in Projection (VIP) was calculated and used a cut-off value of 1.0 to select important features (VIP > 1, FC > 2 or FC < 0.5, *p* < 0.05). Through metabolic enrichment and pathway analysis based on the KEGG database.^1^

## 4 Proteomics analysis

### 4.1 Protein extraction and digestion

Samples were thawed and transferred into lysis buffer (8 M urea, protease inhibitor). Subsequently, the homogenized samples were centrifuged at 12,000 g for 30 min at 4°C to collect the supernatant. The protein concentrations were determined using the BCA method. Protein samples (100 μg) were mixed with lysate supplemented with a final concentration of 100 mM triethylammonium bicarbonate buffer (TEAB). A final concentration of 10 mM TCEP was added, followed by incubation at 37°C for 60 min. Then, a final concentration of 40 mM iodoacetamide was added and allowed to react at room temperature without light for 40 min. The resulting solution was mixed with 6 volumes of cold acetone and incubated again at −20°C for 4 h. Afterward, the solution was centrifuged (at a speed of 10,000 g for 20 min), and the precipitate was collected. To dissolve the sample completely, it was treated with 100 μL of a fully dissolved solution containing TEAB (100 mM). Trypsin was then added in an enzyme-to-protein mass ratio of 1:50, followed by overnight at 37°C.

### 4.2 High pH RP separation

Equal amounts of the digested peptides were mixed and concentrated by vacuum centrifugation. Then the peptides were redissolved by UPLC loading buffer (Thermo Scientific Vanquish F UHPLC, Waltham, MA, United States), and they were then separated using a reverse phase C18 column (ACQUITY UPLC BEH C18 Column 1.7 μm, 2.1 mm × 150 mm, Waters Corporation, Milford, MA, United States) at a high PH. The gradient was set to 3.8–43% mobile phase B for 47 min, with pH adjusted to 10.0 by ammonia water. We collected 20 fractions according to peak pattern and time. Each component was dried in a vacuum concentrator (rotation vacuum concentration, Christ RVC 2-25, Christ, Germany) for the next step. B meant for 80% acetonitrile.

### 4.3 DIA data analysis

The database data was searched and analyzed using the Proteome Discoverer software, and the database search results were then imported into the Spectronaut software to establish a spectrum library. According to the ion pair information of the spectrum library, the Spectronaut software extracted the peak of the DIA raw data. The default settings of the software and iRT correction were used to conduct the analysis. For each protein, up to 6 distinct peptides and up to 6 child ions/per peptide were chosen, and the sum of their areas was used for quantitative analysis. The same quantity of peptide was dissolved in mass spectrometry sample buffer, and 10 × iRT peptide was proportionally added and mixed for DIA detection and analysis. Data acquisition software and gradient, etc. The DDA method is used. The primary mass spectrum resolution is 70,000, the scan range (m/z) is 350–1,300, the AGC target is 3e 6, and the injection duration is 20 milliseconds. Secondary mass spectrum resolution is 17,500; AGC target is 5e 5; injection time is automatic; and HCD; NCE is 28%. The Variable Window is 30, and the Total Cycle Time is 2.85 s.

## 5 RNA isolation and q-PCR validation of gene expression

Using Trizol reagent (Takara Biotechnology, [Dalian] Co. Ltd., China), total RNA was extracted from the frozen duodenum, jejunum, ileum, cecum, colon, and liver by the manufacturer’s instructions. A NanoDrop ND-2000 spectrophotometer (NanoDrop, Germany) was used to measure the purity and concentration of the RNA. All samples were eligible for further analysis since the OD_260_:OD_280_ ratio ranged from 1.8 to 2.0. The extracted and qualified total RNA samples were used as templates to Reverse transcribe and synthesize corresponding cDNA, which was carried out according to the instructions in the GoScript Reverse Transcription System of Promega Corporation (Takara Biotechnology, [Dalian] Co. Ltd., Japan). Using the TB Green^®^ Premix Ex Taq^™^ II kit (Takara Biotechnology, [Dalian] Co. Ltd., Japan), q-PCR was carried out according to the manufacturer’s instructions. The corresponding primers were designed by the PCR primer design principle; all PCR primers were obtained from (Genewiz Biotechnology, [Suzhou] Co. Ltd., China). The mRNA expression levels were normalized to GAPDH and calculated by 2^−ΔΔCt^method. Details of the PCR primers are listed in Supplementary Table S1.

## 6 Statistical analysis

GraphPad Prism 9.0 (GraphPad Software, San Diego, CA, United States, www.graphpad.com) was used for statistical analysis. After conducting the Shapiro–Wilk test and F test on the two sets of data, we proceeded with the t-test. The Mann–Whitney U test was employed to assess data that exhibited non-normal distribution. The average and standard error of the mean (SEM) were used to present the results. *p* < 0.05 was used to identify a significant difference, and *p* < 0.01 indicated a very significant statistical significance. Correlation analysis was conducted using Pearson correlation analysis.

## 7 Results

### 7.1 The influence of delivery modes on hematological parameters of piglets

In the study, two groups of newborn pigs—NF and CS—had their blood physiological and biochemical indices examined. Significant differences were seen in several blood parameters between the two groups. In comparison to the NF group, the CS group showed significantly reduced levels of WBC, NEU, MON, RBC, HGB, and HCT. On the other hand, LYM% and EOS were considerably greater in the CS group (*p* < 0.01, Table 1). The effects of birth mode on five categories of liver function, renal function, blood glucose, blood lipids, and electrolytes were also compared in this study. The results showed that six liver function indices (AST, ALP, AST/ALT, TP, ALB, and GLO), CHOL blood lipid index, and three electrolyte indices (K, NA, and CL) in the NF group were all higher than those in CS group (*p* < 0.01, Table 2). Furthermore, neonatal piglets in the CS group had slightly higher blood glucose levels (GLU), however, these differences were not statistically significant. In essence, cesarean sections appear to hurt liver and renal functions, blood lipid profiles, and electrolyte balance, although the effects on blood glucose levels are less pronounced.

**Table 1:** Impact of natural farrowing and C-section on blood routines of newborn pigs.

Items	Grouping		*p*-value
	NF group	CS group	
WBC, 10^9^/L	7.00 ± 0.64	3.04 ± 0.47	<0.01
NEU%	60.88 ± 5.61	36.69 ± 4.00	<0.01
LYM%	32.93 ± 2.77	48.48 ± 9.26	0.13
MON%	4.71 ± 3.64	11.94 ± 7.01	0.38
EOS%	1.29 ± 0.39	2.10 ± 0.54	0.24
BAS%	0.25 ± 0.04	0.80 ± 0.25	0.04
NEU, 10^9^/L	4.47 ± 0.71	1.14 ± 0.23	<0.01
LYM, 10^9^/L	2.19 ± 0.10	1.59 ± 0.38	0.15
MON, 10^9^/L	0.24 ± 0.16	0.24 ± 0.13	0.99
EOS, 10^9^/L	0.09 ± 0.03	0.06 ± 0.02	0.35
BAS, 10^9^/L	0.02 ± 0.01	0.02 ± 0.01	0.43
RBC, 10^12^/L	6.09 ± 0.32	2.28 ± 0.39	<0.01
HGB, g/L	122.13 ± 7.66	42.50 ± 7.56	<0.01
HCT, %	39.18 ± 2.27	14.66 ± 2.45	<0.01
MCV, fL	64.2 ± 0.72	64.19 ± 1.89	0.99
MCH, pg	19.99 ± 0.36	18.54 ± 0.80	0.11
MCHC, g/L	311.00 ± 3.06	288.43 ± 5.83	<0.01
RDW-C, %	16.35 ± 0.26	16.56 ± 0.40	0.66
RDW-S, fL	39.68 ± 0.64	39.70 ± 1.02	0.98
PLT, 10^9^/L	296.63 ± 40.21	226.00 ± 40.53	0.24
MPV, fL	9.90 ± 0.69	9.53 ± 0.63	0.70
PDW, fL	9.84 ± 0.20	12.13 ± 1.28	0.08
PCT, %	0.29 ± 0.04	0.21 ± 0.03	0.11

Values are means ± SEMs, *n* = 8 per group. NF, natural farrowing; CS, C-section; WBC, white blood cell; NEU%, neutrophil ratio; LYM%, lymphocyte ratio; MON%, monocyte ratio; EOS, eosinophilic ratio; BAS, basophilic granulocyte ratio; EOS, neutrophile granulocyte; BAS, basophilic granulocyte; RBC, red blood cell; HGB, hemoglobin concentration; HCT, hematocrit; MCV, mean corpuscular volume; MCH, mean corpuscular hemoglobin content; MCHC, mean corpuscular-hemoglobin concentration; RDW-C, coefficient of variation of red cell distribution width; RDW-S, standard deviation of red blood cell distribution width; PLT, blood platelet; MPV, mean platelet volume; PDW, platelet distribution width; PCT, platelet volume.

**Table 2:** Impact of natural farrowing and C-section on the blood biochemical parameters of newborn pigs.

Items	Grouping		*p*-value
	NF group	CS group	
ALT, U/L	23.50 ± 3.04	16.00 ± 6.65	0.32
AST, U/L	48.63 ± 15.37	32.63 ± 13.63	0.45
GGT, U/L	161.63 ± 11.07	121.63 ± 20.31	0.11
ALP, U/L	1235.25 ± 87.72	795.50 ± 62.04	<0.01
AST/ALT	2.22 ± 0.73	2.04 ± 0.23	0.82
TP, g/L	25.49 ± 0.81	19.10 ± 1.16	<0.01
ALB, g/L	7.56 ± 0.55	4.35 ± 0.55	<0.01
GLO, g/L	17.93 ± 0.48	14.75 ± 1.01	0.01
A/G	0.44 ± 0.04	0.30 ± 0.04	0.02
CHE, U/L	391.88 ± 18.03	254.50 ± 8.50	<0.01
BUN, mmol/L	3.49 ± 0.37	4.50 ± 0.22	0.04
CREA, μmol/L	108.56 ± 9.73	140.71 ± 13.37	0.07
UA, mmol/L	26.38 ± 2.11	19.25 ± 2.02	0.03
CHOL, mmol/L	1.50 ± 0.10	0.85 ± 0.07	<0.01
TG, mmol/L	0.17 ± 0.03	0.10 ± 0.02	0.08
GLU, mmol/L	4.14 ± 0.54	5.97 ± 1.11	0.16
K, mmol/L	7.25 ± 0.36	5.27 ± 0.28	<0.01
NA, mmol/L	142.11 ± 1.13	143.59 ± 2.17	0.56
CL, mmol/L	107.20 ± 0.66	4.50 ± 0.22	0.01

Values are means ± SEMs, *n* = 8 per group. NF, natural farrowing; CS, C-section; ALT, alanine transaminase; AST, aspartate transaminase; GGT, alanine aminotransferase; ALP, alkaline phosphatase; AST/ALT, aspartate aminotransferase/alanine aminotransferase; TP, total protein; ALB, albumin; GLO, globulin; A/G, ratio of albumin to globulin; CHE, cholinesterase; BUN, blood urea nitrogen; CREA, creatinine; UA, uric acid; CHOL, total cholesterol; TG, triglyceride; GLU, glucose sugar; K, potassium; NA, sodium; CL, chlorine.

### 7.2 Different modes of delivery alter the metabolic profiles of SCFAs and organic acids in newborn piglets

**Figure 1:**
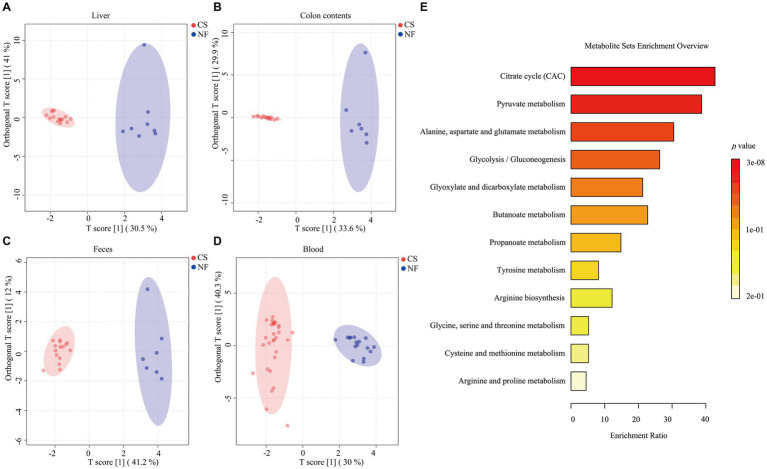
Typical metabolic profiles revealed by OPLS-DA and enrichment of metabolic pathways. **(A–D)** The score plots of the OPLS-DA model between the NF and the CS group in the liver, colon contents, feces, and blood. **(E)** 17 metabolites annotated to the Kyoto Encyclopedia of Genes and Genomes (KEGG) database are displayed in the histogram. Depending on the *p*-value, different colors in the histogram represent the degree of enrichment.

**Figure 2:**
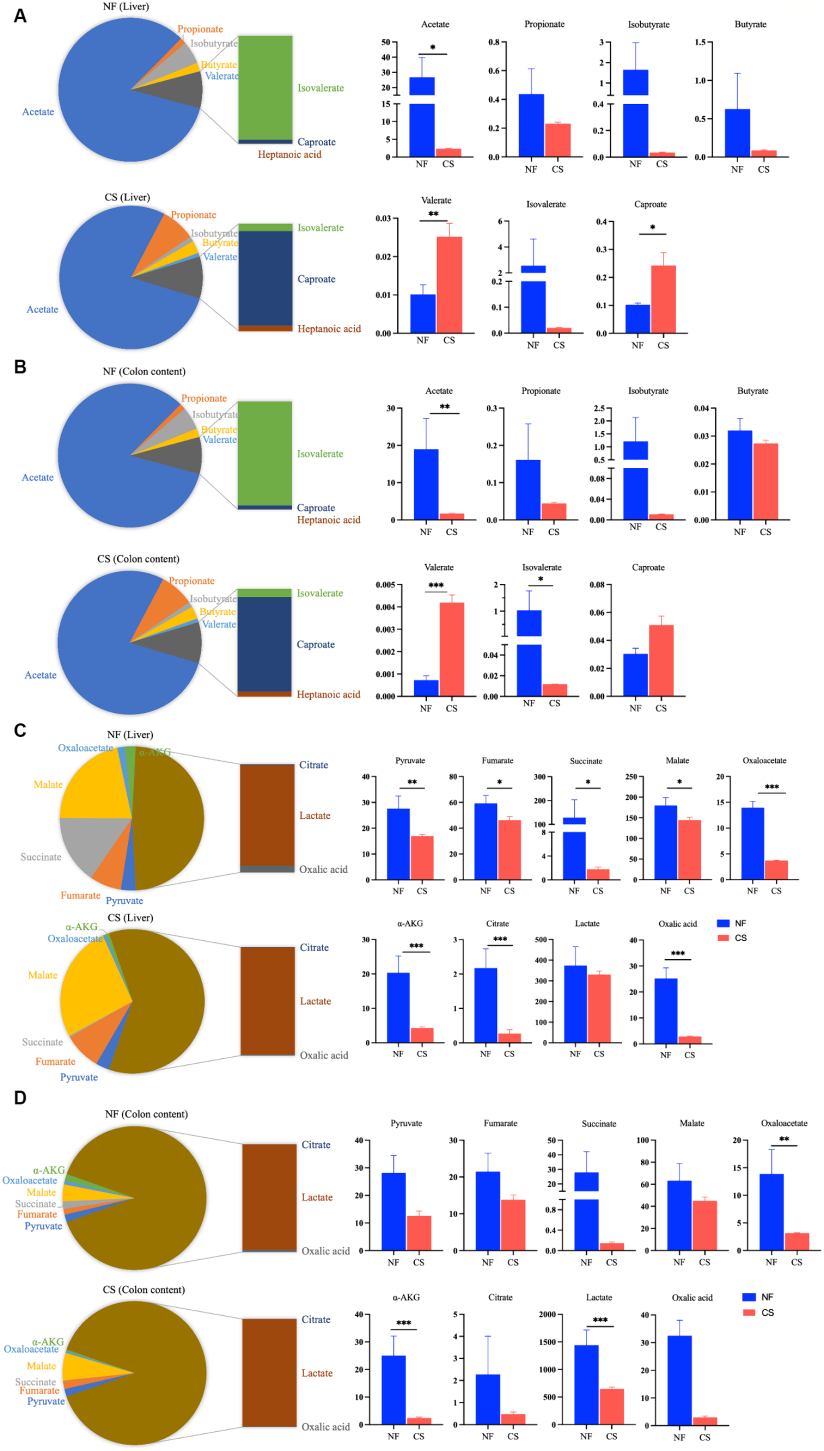
Metabolite profiling of liver tissue and colonic content. **(A,B)** The detection of SCFAs in the liver and colon contents of NF and CS group, and bar graph for between-group analysis of SCFAs concentrations in liver and colon contents. **(C,D)** The detection of organic acids in the liver and colon contents of NF and CS groups, and bar graph for between-group analysis of organic acids concentrations in liver and colon contents.

We analyzed liver, colon, feces, and blood samples from newborn pigs delivered naturally and by C-section using LC/MS/MS to profile 17 metabolites, including 8 SCFAs and 9 organic acids. OPLS-DA modeling distinguished clear metabolic differences between the NF and CS groups across the four tissues (Figures 1A–D), with high Q2 values (>0.85) indicating the model’s predictive accuracy (Supplementary Figure S1). Metabolite contributions to the model were identified, and pathway enrichment revealed that these metabolites were primarily involved in various metabolic processes such as the citrate cycle, pyruvate metabolism, and glycolysis/gluconeogenesis (Figure 1E). Specifically, the NF group’s liver samples were rich in acetate, isovalerate, isobutyrate, butyrate, and propionate, while the CS group had less acetate, isobutyrate, isovalerate and more valerate (*p* < 0.05) (Figure 2A). In colon contents, the NF group had higher levels of acetate, whereas the CS group showed increased valerate and caproate (*p* < 0.05) (Figure 2B). Organic acids in the NF group’s liver were dominated by malate and succinate, whereas the CS group had lower levels of several organic acids, including pyruvate, fumarate, succinate, malate, oxaloacetate, α-AKG and oxalic acid (*p* < 0.05) (Figure 2C). The colon contents of the NF group were marked by lactate, malate, and oxalic acid, while the CS group had lower levels of succinate, oxaloacetate, α-AKG, lactate and oxalic acid (*p* < 0.05) (Figure 2D). In summary, our findings suggest that the mode of delivery significantly influence the metabolic profiles of SCFAs and organic acids in newborn piglets, with C-section births having an impact on the concentrations of key metabolites in liver and colon tissues.

### 7.3 Overview of proteome differences between groups

**Figure 3:**
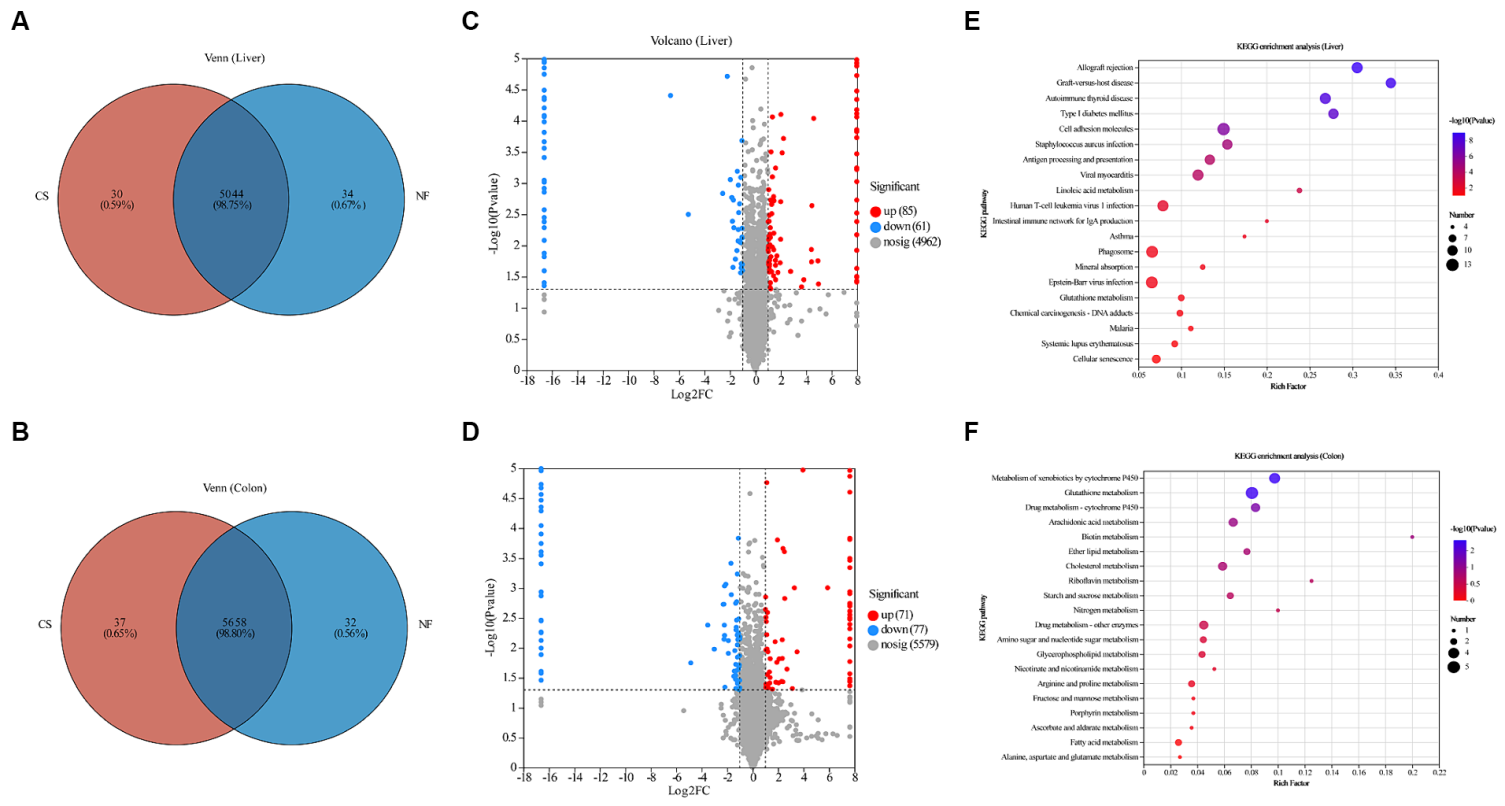
Overview of proteomes of newborn piglet livers and colon, and functional enrichment of DEPs. **(A,B)** Venn diagram of the number of DEPs among liver and colon samples of newborn piglets in CS and NF groups. **(C,D)** Volcano plots of proteomics data from newborn piglets in the CS and NF groups. The *X* axis is log2 (a fold change), and the *Y* axis is −log10 (*p*-value). Each point represents a differential protein. Red dots are proteins that are significantly upregulated, blue dots are proteins that are significantly downregulated in expression, and gray dots are proteins with no significant differences. The dotted lines showed 1 FC and *p* = 0.05. Names of proteins exhibiting significant differences (*p* < 0.05 and log2 FC > 1) between the CS and NF group were shown. Bubble maps showing DEPs in liver **(E)** and colon **(F)** tissues annotated by comparisons to the Kyoto Encyclopedia of Genes and Genomes (KEGG) database. DEPs were classified by KEGG pathway enrichment and significance analysis. The *X*-axis represents the KEGG enrichment fraction; the *Y*-axis represents pathway items. The circle’s color indicates the *p* value and the size indicates the number of DEPs. The process is redder and more prominent, meaning that the enrichment degree of this pathway is higher.

We used DIA proteomic analysis and generated a Venn diagram to compare the difference in protein expression levels in the liver and colon tissues of newborn pigs born through C-section and natural delivery. Our proteomic analysis revealed distinct protein expression patterns in the liver and colon tissues of newborn piglets from C-section and natural deliveries. The liver had 5,044 common proteins, with 34 unique to NF and 30 to CS, while colon tissues showed 5,658 common proteins, 32 unique to NF, and 37 to CS (Figures 3A,B). The liver samples had 146 differentially expressed proteins (DEPs), with 85 up-regulated and 61 down-regulated, and the colon had 148 DEPs, including 71 up-regulated and 77 down-regulated proteins (Figures 3C,D). KEGG pathway analysis indicated that DEPs in the liver were mainly linked to linoleic acid, glutathione, and arachidonic acid metabolism (Figure 3E), while in the colon, they were associated with xenobiotics metabolism, glutathione metabolism, and drug metabolism-cytochrome P450 (Figure 3F). All things considered, these results demonstrate how the birth mode has a major influence on the proteome profile and metabolic pathways in the tissues of newborn piglets.

### 7.4 Association analysis of 17 targeted metabolites and DEPs

**Figure 4:**
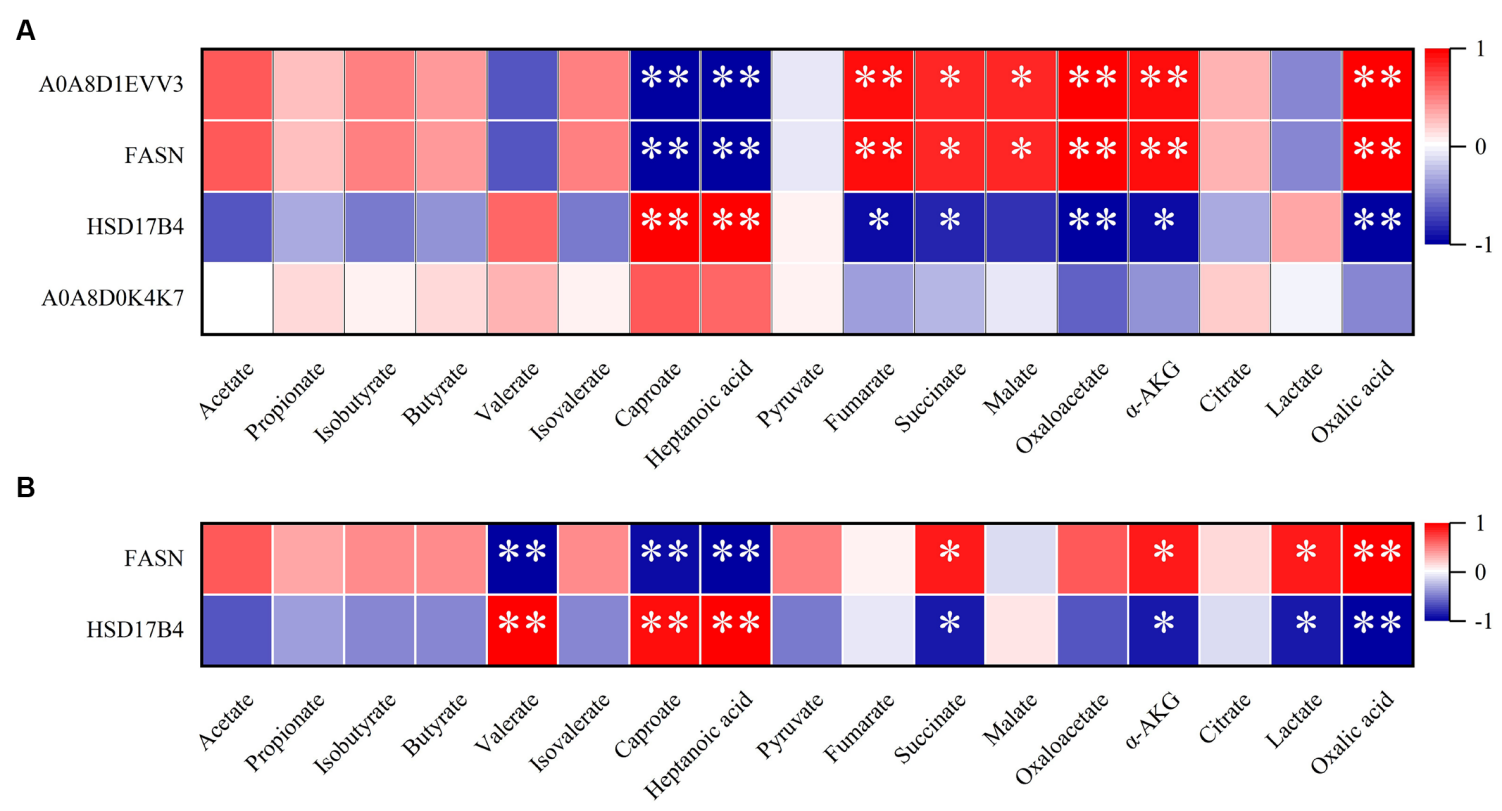
Correlation analysis of DEPs and metabolites in the natural farrowing (NF) group and cesarean section (CS) group. **(A)** Correlation analysis of DEPs and metabolites in CS group and NF group in liver tissue. **(B)** Correlation analysis of DEPs and metabolites in CS group and NF group in colon tissue. The color scale represents the strength of correlation, ranging from 1 (strong positive correlation) to −1 (strong negative correlation); *p ≤ 0.05 and **p ≤ 0.01.

Next, we further conducted clustering analysis on the DEPs in the livers and colons of newborn pigs under different birth modes. We conducted a detailed analysis of DEPs in the liver and colon of newborn piglets from different birth modes, focusing on their relationship with fatty acid metabolism. Pathway enrichment analysis highlighted DEPs involved in fatty acid metabolism, and a correlation analysis was performed with 17 metabolites after log-transforming (log2) the date. In liver tissue, we identified four DEPs that enriched in the fatty acid metabolism pathway and found correlations with 17 metabolites. The results showed that the levels of A0A8D1EVV3 and FASN2 proteins were significantly negatively correlated with the levels of caproate and heptanoic acid in SCFAs. However, they were positively correlated with the levels of fumarate, oxaloacetate, α-AKG, oxalic acid, succinate, and malate. HSD17B4 showed positive correlations with caproate and heptanoic acid, while negative correlations with oxaloacetate, oxalic acid, fumarate, succinate, and α-AKG. Although no significant association was found for A0A8D0K4K7, its overall trend was similar to that of HSD17B4 (Figure 4A). In the same way, in colon tissue, two DEPs, FASN and HSD17B4, were enriched in the fatty acid biosynthesis pathway. Further analysis of 17 metabolites with these DEPs revealed that FASN was negatively correlated with changes in valerate, caproate, and heptanoic acid, while positively correlated with changes in oxalic acid, succinate, α-AKG, and lactate. On the other hand, HSD17B4 showed positive correlations with valerate, caproate, and heptanoic acid, and negative correlations with oxalic acid, succinate, α-AKG, and lactate (Figure 4B). According to the former study, it demonstrated that the significance of FASN as key enzymes in fatty acid metabolism, particularly in certain forms of colon cancer that exhibit overexpression of FASN and its crucial role in the energy metabolism of fatty acids (35, 36).

### 7.5 Analysis of SCFA receptor gene expression

**Figure 5:**
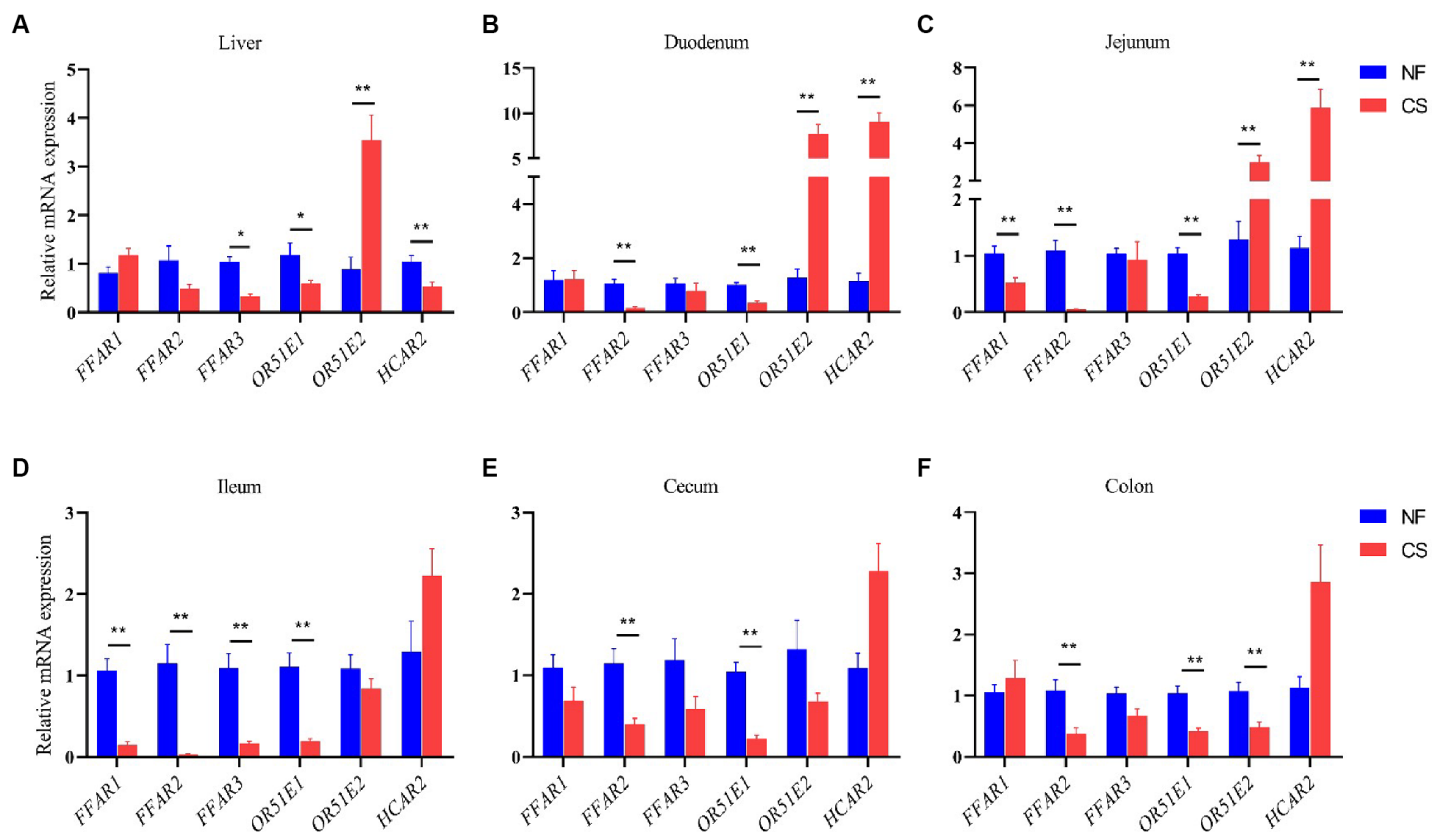
Effects of NF vs. CS on relative mRNA expression levels of *FFAR1*, *FFAR2*, *FFAR3*, *OR51E1*, *ORG1E2*, and *HCAR2*. **(A)** Relative mRNA expression levels in the liver. **(B)** Relative mRNA expression levels in duodenum. **(C)** Relative mRNA expression levels in the jejunum. **(D)** Relative mRNA expression levels in ileum. **(E)** Relative mRNA expression levels in cecum. **(F)** Relative mRNA expression levels in the colon.

SCFAs can co-mediate important cellular processes such as proliferation, differentiation, apoptosis, and immune responses through activation of G protein-coupled receptors (GPCR) (37). In this study, *FFAR1 (GPR40)*, *FFAR2 (GPR43)* and *FFAR3 (GPR41)* were investigated, as well as *OR51E1*, *OR51E2 (Olfr-78)* and *HCAR2 (GPR109A)* genes expression of in liver, duodenum, jejunum, ileum, cecum and colon of newborn piglets in NF and CS groups. In the liver and intestinal segments, the expression of *FFAR2* and *OR51E1* genes was decreased, while the expression of the *HCAR2* gene was increased in intestinal segments (Figure 5). Compared with the NF group, the expression of the SCFAs receptor *FFAR3* in the liver of newborn piglets in the CS group was significantly decreased, there was also a trend toward decreasing acetate, propionate, and butyrate in the CS group, which is in line with the metabolic results. While the expression of the olfactory receptor gene *OR51E2* was significantly up-regulated in the CS group (*p* < 0.01, Figure 5A). In the duodenum and jejunum tissues, the mode of birth by C-section significantly stimulated the expression of *OR51E2* and *HCAR2* genes (*p* < 0.01, Figures 5B,C). In the ileum, the expression levels of *FFAR1*, *FFAR2*, *FFAR3*, and *OR51E1* genes in the CS group were decreased (*p* < 0.01, Figure 5D). Similarly, in the cecum and colon tissues, the expression of *FFAR2*, *OR51E1*, and *OR51E2* genes was decreased by C-section delivery (*p* < 0.01, Figures 5E,F).

## 8 Discussion

While existing literature predominantly focuses on early- and mid-preterm animal models, there is a dearth of studies investigating late-preterm and early-term animal models. Limited research has been conducted thus far regarding the impact of delivery mode on intestinal and liver metabolites (such as SCFAs and organic acids) in late-preterm and early-term newborns, necessitating further accumulation of metabolic data in this population. Using animal models to simulate the physiological development of preterm infants of different gestational ages is a feasible research way to develop a treatment for preterm birth. As one of the large animal models, pigs are especially suitable for pediatric basic research and treatment development of preterm infants, such as intestinal development and diseases, material metabolism, and immune function shaping (38, 39). Our study uniquely focuses on late-preterm and naturally-born piglets, a less explored model in the literature. While previous studies have focused primarily on extremely preterm or early preterm infants, our study fills a gap in the investigation of animal models of late preterm infants (6).

Microbial metabolites play a crucial role in connecting diet, gut microbiota, and host metabolism. They are also important for identifying biomarkers and metabolic therapeutic targets (40). Intestinal SCFAs in humans and mammals are the result of microbial fermentation of dietary fiber and resistant carbohydrates and serve as utilizable metabolites for the host (41). Fecal samples may not fully reflect microbial activity since they do not capture the array of bioactive compounds of microbial origin that are concurrently present in the small intestine (42). However, in the colon, colonic probiotics ferment complex carbohydrates, including dietary fibers and starches, which are indigestible to the host, to produce SCFAs. Systemic SCFAs are metabolized and transported through the liver after absorption and digestion. SCFAs undergo metabolic processes within liver tissue to produce energy and other byproducts. These byproducts are then circulated through the bloodstream to nourish other parts of the body. Recent research suggests that SCFAs produced in the gut can promote liver regeneration through the gut-liver axis (25). Our research focuses on conducting in-depth qualitative and quantitative analysis of SCFAs specifically within the colon and liver regions due to their significance. We found that naturally-born piglets had higher levels of acetate in their liver and gut (26.79 μg/g and 18.93 μg/g) compared to those delivered via C-section, which had lower levels of primary SCFAs. Additionally, C-section pigs had higher levels of trace SCFAs, specifically valerate and caproate, in their liver and colon. The study found that the group born via C-section had higher concentrations of SCFAs in their blood and fecal samples, particularly isobutyrate and butyrate (Supplementary Figure S2). Therefore, the study suggests that relying solely on blood and fecal samples for SCFA measurements may be insufficient. To complement the physiological data of similar neonates, it is necessary to measure SCFA concentrations in the liver and colon of late-preterm piglets born via C-section. Our study’s findings on the impact of delivery mode on the metabolic profiles of SCFAs and organic acids in newborn pigs are consistent with several recent studies investigating the effects of birth mode on gut microbiota and metabolite levels. For example, Wampach et al. (12) showed that the method of birth affects the early functions of the gut microbiome that confer strain and immunostimulatory potential. This is consistent with our findings of altered levels of SCFAs in piglets born by C-section (12). Additionally, Zhou and colleagues (2023) have reported that the transfer of vaginal microbiota can exert an influence on the neurodevelopment and microbiome of infants delivered via C-section. This highlights the importance of delivery mode in shaping the early microbial and metabolic environment (16). Furthermore, germ-free mice that lack gut microbiota have lower yet detectable SCFA levels, particularly acetic acid, in their gut and peripheral tissues compared to conventional mice (43).

After being absorbed by the intestines, SCFAs can have an impact on the host’s energy metabolism by entering the host cell’s citric acid cycle (CAC) (28). As a result, the present study also assessed physiological changes in the concentration of intermediate metabolites (the 9 organic acids) of the CAC in the blood, feces, liver, and colon of newborn pigs under two delivery modes. Lactic acid is the highest concentration of organic acid in newborn pigs in the NF group, and higher than that in the CS group. It is known that in mammalian tissues, glucose can be metabolized into lactate through anaerobic decomposition, and circulating lactate can also be the main substrate of CAC “carbon” to provide an energy source, especially when the animal is fasting, the contribution of circulating lactate to CAC intermediates exceeds glucose (44). The latest study confirmed that the function of the CAC 2.1 complex is to balance the stress situation within the cell (45). Therefore, we have reason to speculate that the physiological significance of higher concentrations of lactic acid produced in the tissues of newborn piglets in the natural production group is likely to be triggered by activating the CAC 2.1 complex to trigger the following biosynthetic pathways: lactic acid → CAC 1.1 → malic acid → CAC 2.1 → succinic acid, accelerating the recovery of ATP in tissue cells, to promote faster nutrient supplementation of tissue cells. α-AKG, an intermediate of the CAC derived from glutamine, has been proposed as the key factor determining glutamine dependence in cells (46). Studies have demonstrated the significant role of the gut microbiome in the development of newborns’ gut mucosal barriers (47). In preterm neonates, necrotizing enterocolitis (NEC) is primarily caused by intestinal barrier immaturity, as indicated by increased intestinal permeability (48). Despite this understanding, it is still unclear how the different modes of delivery may affect the intestinal barrier function of neonates. Therefore, further investigation is necessary. In this study, we carefully examined proteomic data derived from the colon, with a focus on evaluating the expression levels of crucial proteins involved in forming intestinal tight junctions. This includes both the Zonula occludens (ZO) protein family and the Claudin family. This study aims to compare the effects of C-sections and natural birth on the integrity of the intestinal barrier in newborn piglets. The results showed that the mode of delivery did not significantly affect the intestinal barrier integrity of the piglets (*p* > 0.05, Supplementary Figure S3). In the context of our study, this finding suggests that the method of delivery may not be the primary determinant of intestinal barrier function in neonatal piglets. Of course, it should be noted that these results interfered with some force majeure factors, such as the operation techniques of the C-section operator or the environmental factors of the cesarean section operation room. There are also current studies that show that during the process of vaginal delivery, newborns are colonized by the microbiota present in the mother’s vaginal and fecal flora. Conversely, in the case of C-section deliveries, the colonization of newborns is influenced by the microbiota found in the hospital environment (49). After analyzing 17 metabolites, 9 differential metabolites were identified (Supplementary Figures S4A–D). Enrichment analysis revealed that in addition to the CAC pathway, there was a pyruvate metabolism pathway (Supplementary Figure S4E). Therefore, according to the results of this study, it is speculated that the mode of birth does affect the metabolism of SCFAs in newborn piglets, and it may be through the influence of the CAC and pyruvate metabolism pathway to change the concentration of cycling intermediates including lactate, malate, etc., and acetate and other SCFAs. These altered concentrations of intermediates and SCFAs are ultimately reflected in physiological differences in energy metabolism in newborn piglets. In short, small molecule metabolites play important functions throughout the body of preterm infants and should therefore be favorable targets for various disease biomarkers in future studies.

FASN and HSD17B4 are DEPs enriched in the fatty acid biosynthesis pathway and fatty acid biosynthesis pathway in colon tissue, respectively, and the expression of FASN in the CS group is significantly down-regulated, HSD17B4 expression was significantly up-regulated in the CS group. It is worth noting that our research revealed a noteworthy correlation between FASN and the dynamic fluctuations in the levels of CAC intermediates, such as oxalic acid, succinate, α-AKG, and lactate, following a C-section delivery. The concentration of these CAC intermediates decreased in the CS group. The findings indicate that the impact of birth mode on the fat metabolism and CAC of piglets becomes evident in the energy recovery observed in full-term pigs at birth, as opposed to late preterm pigs, and naturally born piglets, as opposed to cesarean piglets. These observations imply that there exist physiological disparities in the lipid production capacity between these different groups (50). In the CAC, intermediates are necessary for the synthesis of lipids by FASN (51). In bladder cancer, FASN is a key regulator of fatty acid metabolism and is associated with immunotherapy. In addition, immune analysis showed that FASN was significantly associated with several immune cells (52). Another enzyme, HSD17B4, is also involved in the synthesis and elongation of unsaturated fatty acids (53). This finding highlights the potential influence of FASN on metabolic processes in individuals born via C-section. The association between FASN and the concentration changes in these metabolites suggests a possible role of FASN in modulating metabolic pathways and warrants further investigation.

G protein-coupled receptors (GPCRs) are seven transmembrane receptors that activate the heterotrimeric G protein. Despite extensive research, approximately 150 types of orphan GPCRs remain uncharacterized and have not been explored as potential targets for drug development (54). According to our research findings, the expression of OR51E1 in the liver and various intestinal segments was notably higher in the natural delivery group compared to the C-sections group. *OR51E1* is a subtype of GPCR, and its expression level is known to be linked to the endocrine activity of the gastrointestinal (GI) tract in pigs. Several factors, such as age, pathogens, and diet control, can influence the expression of *OR51E1* in GI tissues (55). *OR51E2*, another olfactory receptor, is activated by SCFAs like acetate and butyrate. The transcription of *OR51E2* is significantly reduced in mouse models with DSS-induced colitis (56). Based on our findings, it seems that C-section delivery hurts the intestinal health of newborn piglets. Moreover, it is worth noting that HCAR2 functions as a butyrate receptor, exhibiting high expression levels in human adipose tissue and spleen while being minimally expressed in the liver (57). Our investigations have revealed that HCAR2 expression in liver tissue is low and that its expression is significantly reduced by C-section birth mode. Conversely, HCAR2 was found to be highly expressed in all intestinal segments, with the CS group exhibiting higher expression levels than the NF group. Activating *HCAR2*, the receptor for niacin and butyrate, suppresses colonic inflammation and carcinogenesis, according to the former study (58). We hypothesized that the C-section might have triggered the activation of *HCAR2*, resulting in its increased expression across different sections of the intestines. It is crucial to acknowledge the findings of previous research, which have demonstrated that *FFAR2*^−/−^ mice display more severe symptoms of colitis (59). *FFAR2* plays a significant role in regulating ILC3-derived IL-22 through AKT and STAT3 signaling, ultimately contributing to the improvement of gut health (60). Our study results indicate a significant down-regulation of *FFAR2* expression in the colon of the CS group, aligning with the outcomes observed in the metabolome targeted by SCFAs.

## 9 Conclusion

Pigs serve as an important animal model for studying neonatal intestinal development, particularly in preterm infants. Investigating the impact of delivery methods on neonatal development is a significant area of research in neonatology. In this study, a combined approach using metabolomics and proteomics was employed to determine how birth mode alters the physiological concentrations of SCFAs and organic acids in the liver, colon, feces, and blood of newborn piglets. These alterations may be achieved through changes in the physiological levels of intermediate metabolites in the CAC, including lactate and malate, via the pyruvate metabolic pathway. Different birth modes also resulted in differential protein expression, such as FASN and HSD17B4. Furthermore, there was a certain correlation between these differentially expressed proteins and the dynamic changes in metabolites. Overall, these findings have the potential to shed light on the challenges faced in the clinical application of late preterm infants.

## Data availability statement

The original contributions presented in the study are publicly available. This data can be found at: https://ngdc.cncb.ac.cn/omix; OMIX005286.

## Ethics statement

The animal study was approved by the Institutional Ethics Committee of the Chongqing Academy of Animal Sciences (Chongqing, China) reviewed the relevant ethical issues and approved this study (permit number xky-20150113). All of the experiments were conducted at the Experimental Swine Engineering Center of the Chongqing Academy of Animal Sciences (CMA No. 162221340234; Chongqing, China). The study was conducted in accordance with the local legislation and institutional requirements.

## Author contributions

JC: Conceptualization, Data curation, Software, Writing – original draft, Writing – review & editing. JW: Data curation, Writing – review & editing. HZ: Data curation, Project administration, Writing – review & editing. JZ: Writing – review & editing. YD: Project administration, Supervision, Writing – review & editing. LG: Project administration, Supervision, Writing – review & editing, Funding acquisition, Resources. JM: Project administration, Supervision, Writing – review & editing. JS: Conceptualization, Funding acquisition, Investigation, Methodology, Project administration, Resources, Supervision, Writing – review & editing.

## Footnotes

^1^ http://www.Genome.jp/kegg/
