# Supplementary material for: Exploring functional metabolites and proteomics biomarkers in late-preterm and natural-born pigs
Source: Front Vet Sci. 2024 Apr 24;11:1340849. doi: 10.3389/fvets.2024.1340849 (PMC11076856; doi:10.3389/fvets.2024.1340849)
Supplement: Supplementary file 1 [file Data_Sheet_1.docx]

Supplementary Material

**Exploring functional metabolites and proteomics biomarkers in late-preterm and natural-born pigs**

**Jie Chong^1,2^, Juan Wang^1,2^, Hang Zhong^1^, Jinwei Zhang, Yuchun Ding^1,3^, Liangpeng Ge^1,3^, Jideng Ma^2^, Jing Sun^1,3*^**

^1^ Chongqing Academy of Animal Sciences, Chongqing 402460, China

^2^ Farm Animal Genetic Resources Exploration and Innovation Key Laboratory of Sichuan Province, Sichuan Agricultural University, Chengdu 611130, China

^3^ National Center of Technology Innovation for Pigs, Chongqing 402460, China

*** Correspondence:** Jing Sun: [sunjing85026@163.com](mailto:sunjing85026@163.com)

# Supplementary Figures and Tables

**1.1 Supplementary Tables**

Primer Premier 5.0 was used to design primers, and GAPDH was selected as the reference gene to verify the expression level. By BLAST (https://blast.ncbi.nlm.nih.gov/Blast.cgi) test primer amplification specificity, and the gold extreme intelligence (Genewiz Biotechnology, [Suzhou] Co. Ltd, China) synthesis, primer information table are shown in Table S1.

Supplementary Table 1 Primer sequence

| **Gene** |  | **Primer sequences 5’ to 3’** | **Primer length (bp)** | |
| --- | --- | --- | --- | --- |
| FFAR1 | Forward | GCCTAGGACCCTACAATGCC | | 20 |
|  | Reverse | GATGGGCCCCCTTTTGTTCT | | 20 |
| FFAR2 | Forward | ACGAAGGAGAGCCCCAAATG | | 20 |
|  | Reverse | TGGTGAAGTCAGAACTCGGC | | 20 |
| FFAR3 | Forward | CCGAGTGGAGACCTTACGTG | | 20 |
|  | Reverse | TTCTTCAGTTTCCCGCTGCT | | 20 |
| OR51E1 | Forward | CGCGTCAACATCATCTATGGC | | 21 |
|  | Reverse | CGCACACATGGGAGACACAC | | 20 |
| OR51E2 | Forward | TCCGTGCCAGCTATGAGTTC | | 20 |
|  | Reverse | GCCGATCCAGAAGTGAGCTT | | 20 |
| HCAR2 | Forward | GGCATCTTGTGGGGAATGGA | | 20 |
|  | Reverse | AACTCTCCGGCAGCAGAAAA | | 20 |
| GAPDH | Forward | GCCATCACTGCCACCCAGAA | | 20 |
|  | Reverse | GCCAGTGAGCTTCCCGTTGA | | 20 |

**1.2 Supplementary Figure**


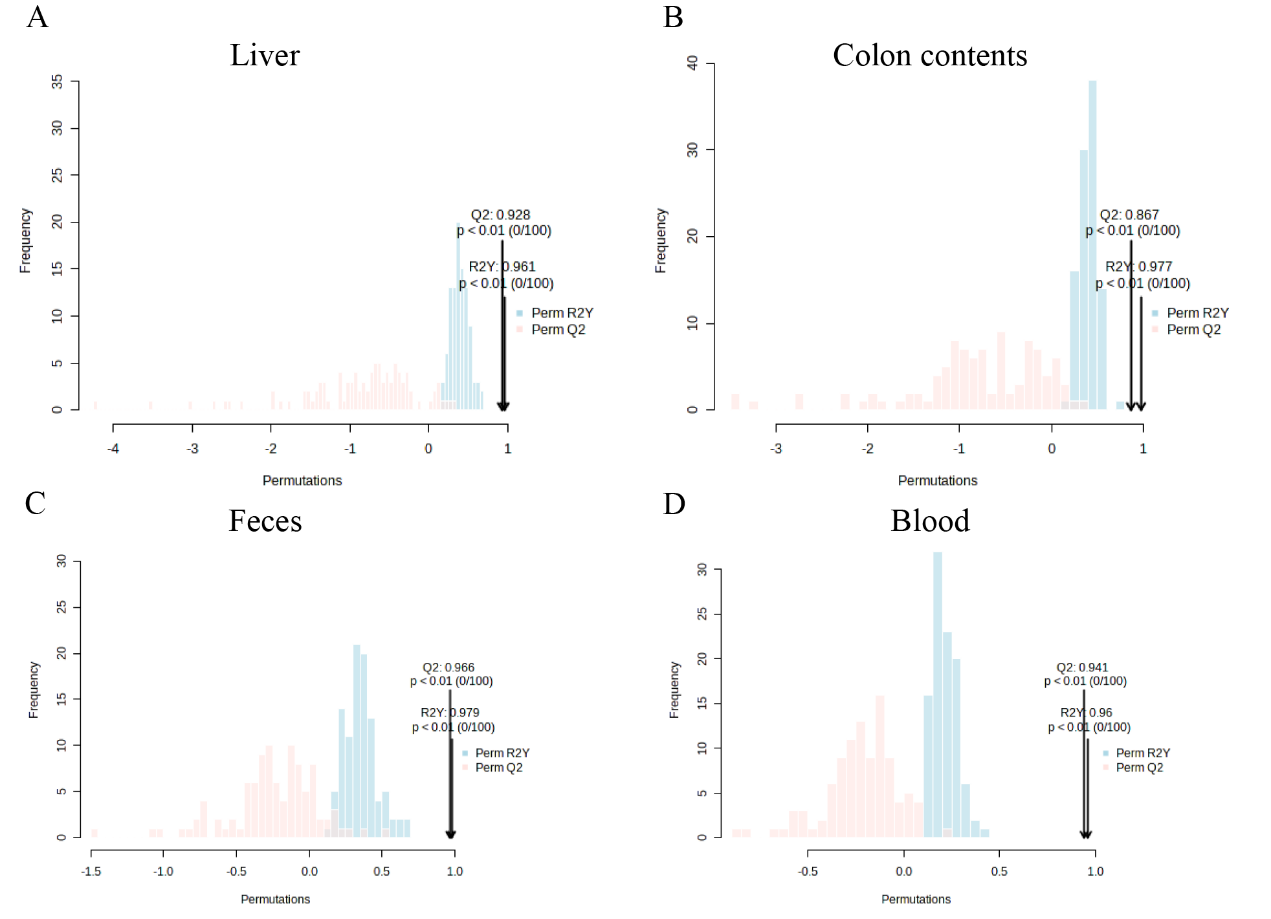


Figure.S1 Permutation tests of OPLS-DA discrimination models between NF and CS. **(A)** The score plots of the OPLS-DA model between NF group and CS group in the liver, with R2Y (cum) =0.961 and Q2 (cum) =0.928. **(B)** The score plots of the OPLS-DA model between CS group and NF group in the colon contents, with R2Y (cum) =0.867 and Q2 (cum) =0.867. **(C)** The score plots of the OPLS-DA model between NF group and CS group in the feces, with R2Y (cum) =0.979 and Q2 (cum) =0.966. **(D)** The score plots of the OPLS-DA model between NF group and CS group in the blood, with R2Y (cum) =0.96 and Q2 (cum) =0.941.


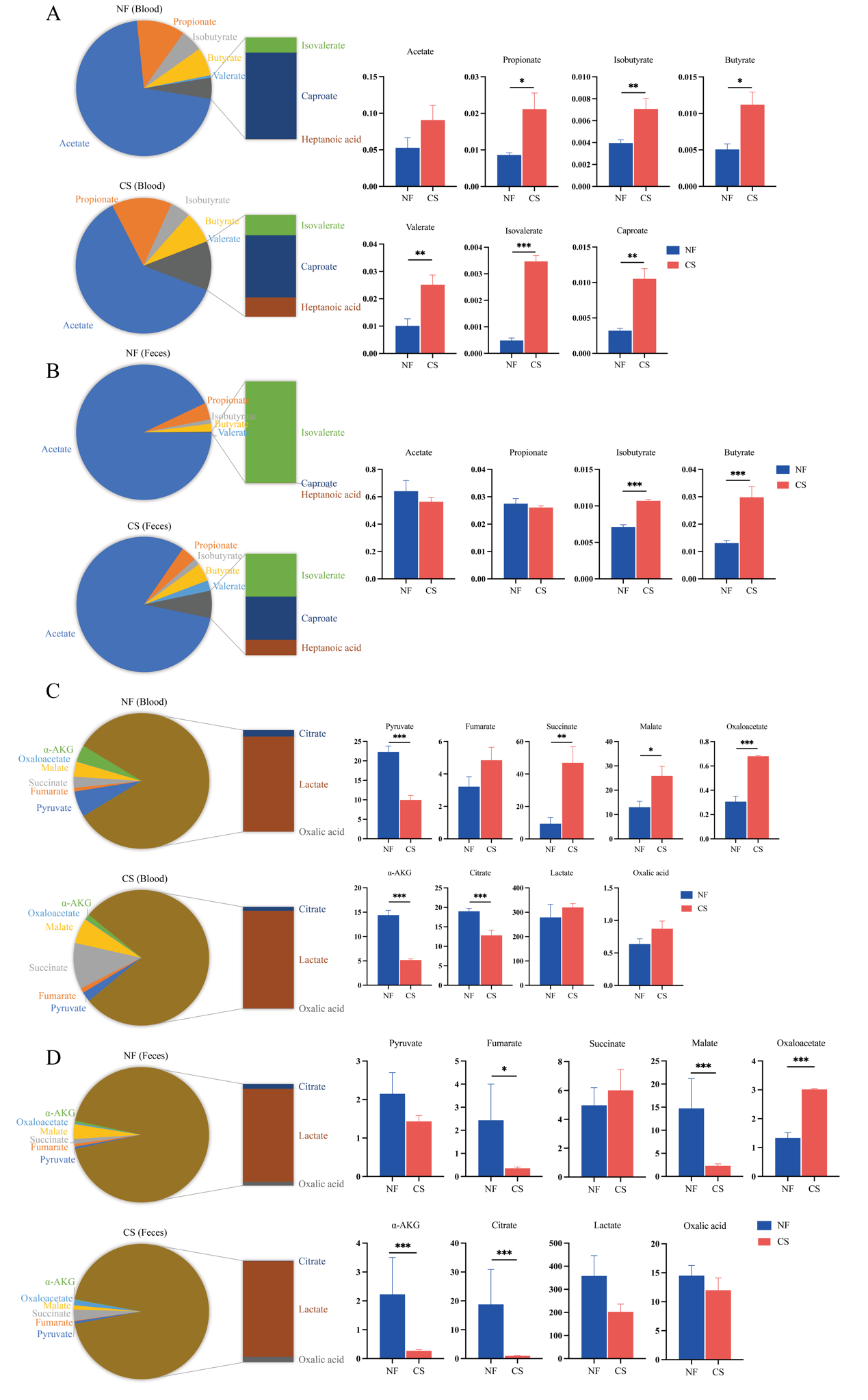


Figure.S2 Metabolite profiling of blood and feces. **(A, B)** The detection of SCFAs in the blood and feces of NF and CS group, and bar graph for between-group analysis of SCFAs concentrations in blood and feces. **(C, D)** The detection of organic acids in the blood and feces of NF and CS groups, and bar graph for between-group analysis of organic acids concentrations in blood and feces.


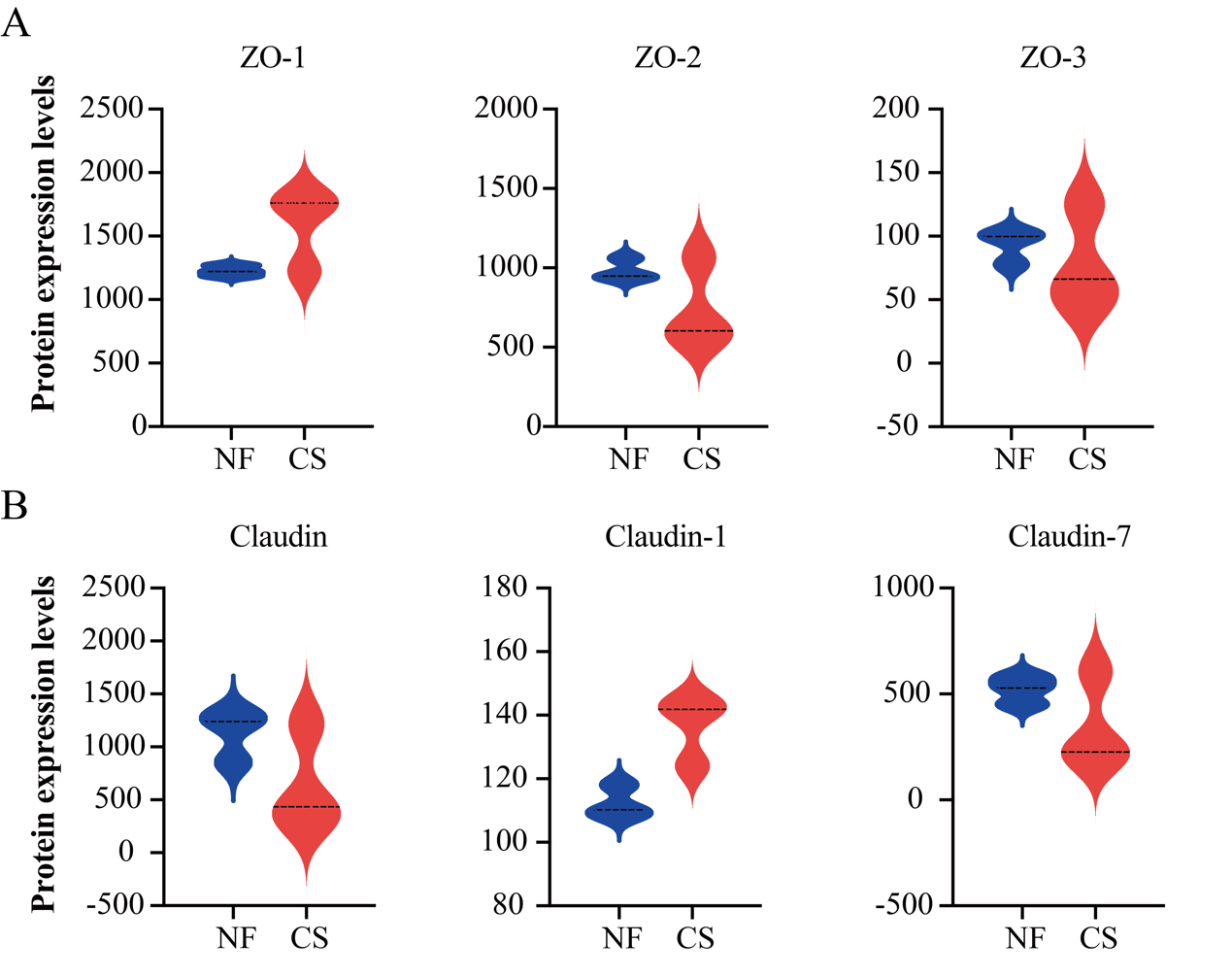


Figure.S3 The expression levels of intestinal tight junction proteins. **(A)** The expression levels of proteins in the ZO protein family, including ZO-1, ZO-2, and ZO-3. **(B)** The expression levels of the Claudin protein family, including Claudin, Claudin-1, and Claudin-7.


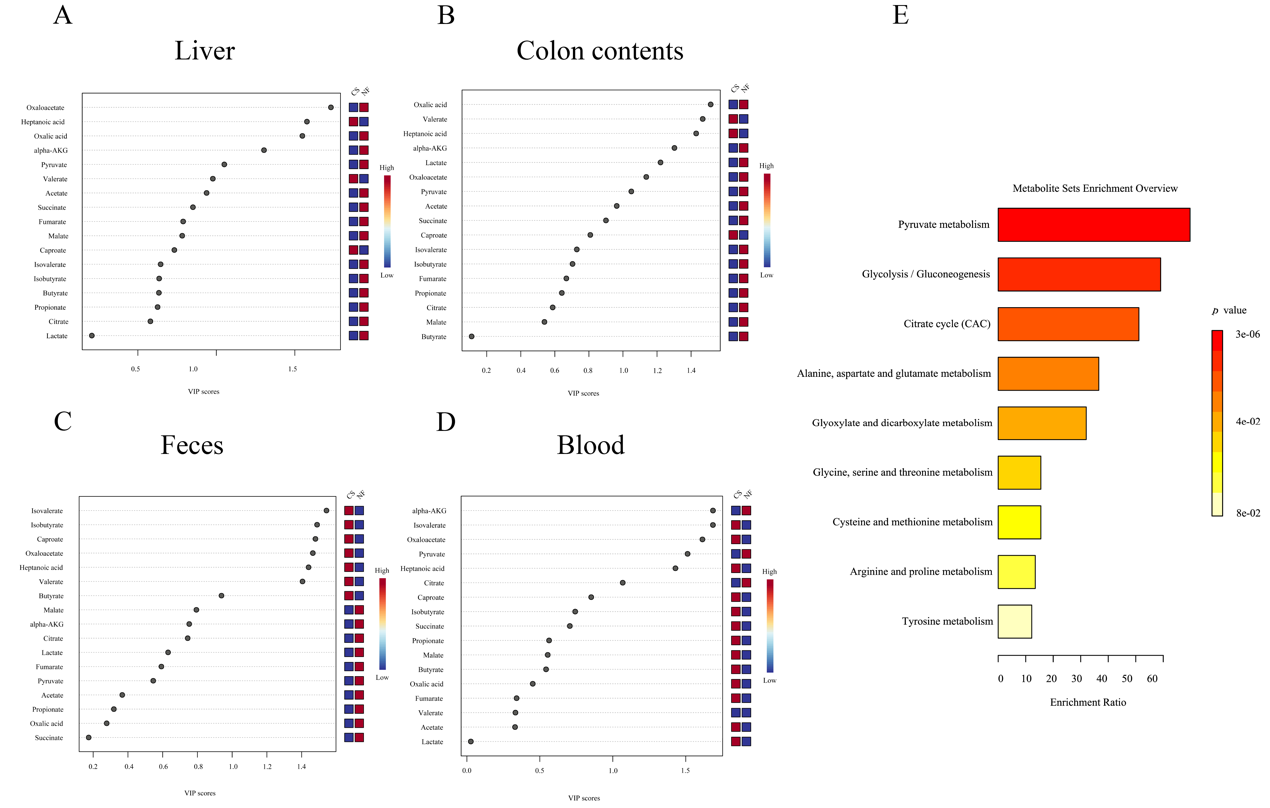


Figure.S4 Differential metabolite selection and enrichment analysis of metabolic pathways. **(A-D)** VIP (Variable important in projection) revealed the metabolites for NF vs CS in liver, colon contents, feces and blood samples. Red is high-expression; blue is low-expression. **(E)** Histogram showing 9 differential metabolites annotated to the Kyoto Encyclopedia of Genes and Genomes (KEGG) database. The degree of enrichment is indicated by different colors in the histogram, according to the *p value*.


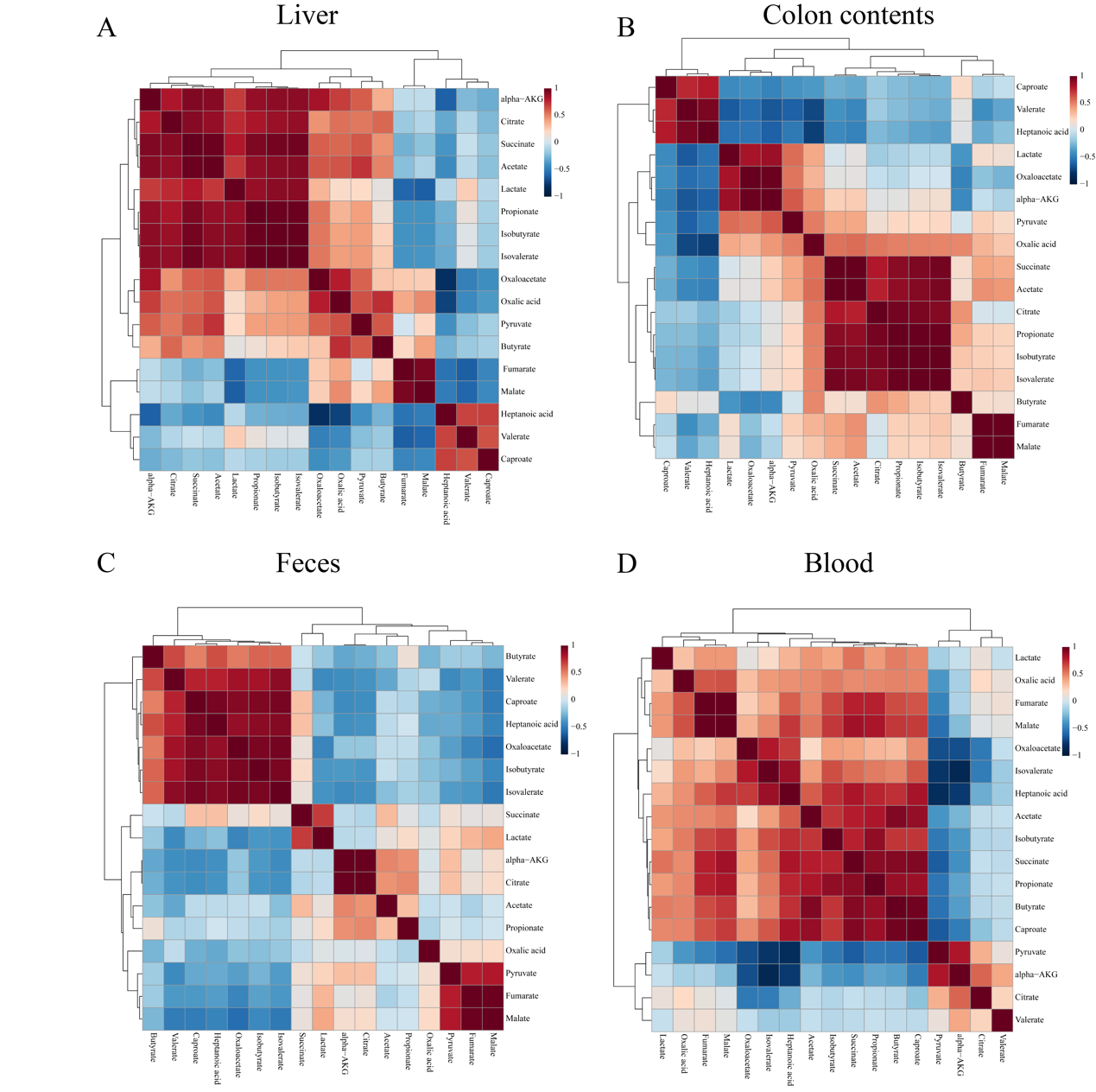


Figure.S5 Metabolite-metabolite correlation analysis in liver, colon contents, feces and blood samples. **(A)** Metabolite-metabolite correlation analysis in liver. **(B)** Metabolite-metabolite correlation analysis in colon. **(C**) Metabolite-metabolite correlation analysis in feces. **(D)** Metabolite-metabolite correlation analysis in blood. Every square represents the Pearson’s correlation coefficient for a pair of metabolites. Red indicates positive relationship, blue indicates negative relationship, and white represents non-significant correlation. The darker the dot is, the higher the degree of correlation between metabolites.
